# Centrifugation-Assisted Ultrafiltration as an Innovative Methodology to Enhance Phenolic Compound Bioaccessibility and Bioavailability from Winery By-Product Extracts

**DOI:** 10.3390/foods15010141

**Published:** 2026-01-02

**Authors:** Juan Antonio Nieto, Laura Jaime, Marin Prodanov, Susana Santoyo

**Affiliations:** Institute of Food Science Research (CIAL), Universidad Autónoma de Madrid (CEI UAM+CSIC), 28049 Madrid, Spain; laura.jaime@uam.es (L.J.); marin.prodanov@uam.es (M.P.); susana.santoyo@uam.es (S.S.)

**Keywords:** ultrafiltration, separation, phenolic compounds, antioxidant, bioaccessibility, bioavailability, by-products, winery

## Abstract

An innovative methodology based on a centrifugation-assisted ultrafiltration process (CUF) has been investigated as a suitable methodology to enhance the bioavailability of phenolic compounds with antioxidant activity from winery by-products. For this purpose, seed (GSE) and stem (STE) extracts obtained by pressurized liquid extraction were processed by applying CUF methodology, generating a seed and stem permeate (PGSE and PSTE, respectively). The evaluated methodology allowed for the removal of the polymeric proanthocyanidin fraction. Thus, PGSE and PSTE resulted in a lower number of phenolic compounds and antioxidant activity compared to GSE and STE extracts. However, meanwhile, the low-molecular-weight fraction showed a close trend in its phenolic profile composition, the quantity of the compounds was increased because of a concentration effect in the permeates. Phenolic compounds bioavailability was conducted through an in vitro static digestion method followed by in vitro intestinal absorption using a Caco-2 cell monolayer model. PGSE and PSTE bioaccessibility was greater than STE and GSE because of an intense loss of the polymeric fraction during the digestion process. In addition, higher amounts of total phenolic compounds, as well as low-molecular-weight phenolics, were determined in the PGSE and PSTE bioaccessible fractions. Furthermore, higher antioxidant and total phenolic compounds were detected in the bioavailable fraction after in vitro intestinal absorption assays for the permeates. Hence, CUF methodology resulted as a suitable and effective technique to enhance the phenolic extracts’ bioavailability, although the phenolic matrix effect should be tested.

## 1. Introduction

In the context of a more sustainable food industry, great efforts have been made to reduce the food waste being generated and improve its value and utilization [1]. In this regard, vegetable by-products have been designed as suitable sources for the extraction and production of rich phenolic compound extracts [2]. Phenolic extracts are considered valuable industrial products, as they can be used as additives in the food industry or, more frequently, as functional ingredients [3,4]. In this context, winemaking by-products have traditionally been used for animal feed [5] or as waste-based compost [6], but in recent decades, they have emerged as suitable sources for obtaining and producing bioactive phenolic compound extracts [7]. Diverse potential health benefits, such as antioxidant capacity [8], anti-inflammatory activity [9], or neuro-protective capacities [10], among others, have been described for these phenolic extracts. However, when considering the potential benefits, the bioaccessibility and bioavailability of the contained bioactive phenolic compounds must be considered, since they comprise a critical step in the bioactivity of the extract [11,12]. In this regard, phenolic compounds’ bioavailability are dependent on several factors, including matrix composition, digestibility, stability, and intestinal absorption, and recently, the phenolic matrix has also been suggested as a factor [11,12,13]. Consequently, a great variability of bioaccessibility or bioavailability results can be found in the scientific literature, even when the same phenolic sample matrix is compared [11,12,14]. In line with this, recent studies suggest a significant matrix and phenolic matrix effect on phenolic compound bioavailability, with a critical role for polymeric procyanidins [11]. These compounds are non-bioavailable, causing intense losses rates during stomach and intestinal digestion, and entailing low bioavailability and great antioxidant reductions in winemaking by-product extracts [11,13,15]. In addition, procyanidin is associated with astringency [16], hindering the organoleptic acceptance of the phenolic extracts, whereas higher concentrations of low molecular flavan-3-ols are required to generate astringency [17]. Thereby, organoleptic issues are also important and should be considered during the development of functional ingredients from winery by-products.

Different methodologies have been investigated with the aim of enriching the total phenolic content of extracts or improving their functional properties, such as post-harvest UV-B irradiation [18], resin treatments for concentration [19], ultrasound-assisted osmotic treatments [20], ultrafiltration, or nanofiltration [21]. Among them, membrane separation processes are useful technologies for recovery, fractionation, and concentration of phenolic compounds [21,22]. Compared to conventional methods, membrane-based technologies offer several advantages, including operation at low temperature, high separation efficiency, low energy consumption, simplicity of equipment, and easy scale-up [23]. The separation mechanisms involved in membrane processes are based on size exclusion. According to the membrane pore sizes, the process can be classified as microfiltration (0.1–5 µm, 1–10 bar), ultrafiltration (0.5–100 nm, 1–10 bar), nanofiltration (0.5–10 nm, 10–30 bar), or reverse osmosis (<0.5 nm, 35–100 bar) [24]. Ultrafiltration membrane has been characterized by a zero rejection of low-molecular-weight compounds such as sugar and salt [25], as well as low-molecular-weight phenolic compounds [21,22], while high-molecular-weight solutes are rejected [25]. However, interactions of phenolic compounds with the different membrane materials must be considered and investigated since they condition the membrane fouling and, therefore, the membrane retention [24]. Additionally, alternative processes with interesting results have been developed through the combination of different membrane unit operations and conventional separation technologies, such as adsorption, centrifugation, or evaporation [26]. In line with this, centrifugation-assisted ultrafiltration (CUF) has been used to characterize low-molecular-weight compounds in phenolic extracts [27]. Nevertheless, no investigations dealing with this specific ultrafiltration process as a technique to obtain functional ingredients enriched in low-molecular-weight phenolic compounds have been reported.

The aim of the present study was to evaluate the potential of centrifugation-assisted ultrafiltration process (CUF) as a low-molecular-weight phenolic compound purification and concentration process to enhance the bioaccessibility and bioavailability of phenolic extracts. To our knowledge, this process has not been used for this purpose before, being generally conducted by a conventional ultrafiltration process. The bioaccessibility and bioavailability of two pressurized liquid extracts (PLEs) obtained from grape stems and seeds were compared with those of their respective permeates obtained using the CUF process. Thus, total phenolic content, polymeric and individual low-molecular-weight phenolic composition, and antioxidant activity of the original PLE stem and seed extracts were monitored and compared during in vitro gastrointestinal digestion and in vitro intestinal absorption with their respective permeates.

## 2. Materials and Methods

### 2.1. Chemicals and Reagents

Acetonitrile and formic acid, HPLC quality, were supplied by Labscan (Dublin, Ireland) and Acros Organic (Geel, Belgium), respectively. Protocatechuic acid, vanillic acid, syringic acid, 3-coumaric acid, ethyl gallate, 3,4′,5-trihydroxystilbene-3-*O*-D-glucoside (*trans*-piceid), (+)-catechin, (−)-epicatechin, epicatechin gallate, procyanidin A2, procyanidin B1, procyanidin B2, quercetin-3-*O*-galactoside, quercetin-3-*O*-rutinoside, quercetin-3-*O*-glucuronide, quercetin-3-*O*-glucoside, and quercetin dehydrate were purchased from Extrasynthèse (Genay, France). Gallic acid, 4 hydroxybenzoic acid, caftaric acid, ellagic acid, *trans*-resveratrol, 6-hydroxy-2,5,7,8-tetramethylchromane-2-carboxylic acid (Trolox), potassium persulfate, 2,2′-azinobis(3-ethylbenzothiazoline-6-sulphonic acid) diammonium salt (ABTS), 2,2-diphenyl-1-picrylhydrazyl (DPPH), phosphate buffer 1 M, fluorescein sodium, 2,2′-azobis(2-methylpropionamidine) dihydrochloride (AAPH), 3-(4,5-dimethylthiazol-2-yl)-2,5-diphenyl tetrazolium bromide (MTT), NaHCO_3_, trizma, and maleate were obtained from Sigma-Aldrich (Madrid, Spain). Disodium carbonate, Folin–Ciocalteu reagent, methanol, and ethanol were from Panreac (Barcelona, Spain).

### 2.2. Plant Material

Grape stems and seeds (*Vitis vinifera* L. cv. Merlot) were provided by Instituto Madrileño de Investigación y Desarrollo Rural, Agrario y Alimentario (IMIDRA, Spain). Grape stem and seed samples were prepared according to previous investigations reported by our group [8]. The vegetable material was stored at −20 °C until further use for extraction processes.

### 2.3. Pressurized Liquid Extraction (PLE)

The PLE from grape seeds (GSE) and grape stems (STE) used in this research were obtained in previous research conducted by our group [8,11]. ASE 350 equipment from Dionex Corporation (Sunnyvale, CA, USA) was used. Briefly, STE was carried out at 120 °C for 10 min with 30% water:ethanol as solvent, whereas grape seeds were extracted at 20 °C, 11 min, and 75% ethanol. The extracts were recovered in glass vials, and ethanol was removed by evaporation (IKA RV 10, IKA, Barcelona, Spain). Then, both extracts were lyophilized in Telstar Lyobeta 15 equipment (Telstar, Barcelona, Spain), and the generated dry powder was kept at −20 °C until further use.

### 2.4. Centrifugation-Assisted Ultrafiltration Process (CUF)

A specific ultrafiltration device consisting of a 10 kDa ultrafiltration membrane coupled to a centrifuge tube (Merk, Millipore, Germany) was used to apply the CUF process to the STE and GSE samples. The membrane chamber of the devices had a capacity of 15 mL and consisted of a two-sided membrane made of regenerated cellulose, with a pore size of 10 kDa. For the ultrafiltration process, the membrane was placed perpendicular to the direction of the centrifugal force. The samples were processed in triplicate, for which the centrifugation devices were single-use. For each CAU treatment, 12 mL of sample (30 mg/mL) diluted in methanol:water (1:1) was placed in the membrane module and centrifuged at 20 °C and 5000 rpm until the extract was completely filtrated (approximately 4 h). Thus, a fraction was retained on the selective side of the ultrafiltration membrane, while a stem extract permeate (PSTE), or a seed extract permeate (PGSE), passed through the membrane and was collected inside the centrifugation tube. After that, methanol was removed from the permeates by vacuum evaporation. Free methanol permeates were lyophilized, and the resulting powder was stored at −20 °C for further analysis. Finally, the resulting final lyophilizates were combined to generate permeate samples.

### 2.5. Identification and Quantification of Phenolic Compounds by RP-HPLC

Phenolic composition of samples was determined by the chromatographic method previously reported by our group [28]. Agilent Infinity 1260 liquid chromatograph equipment, coupled with a photodiode array detector (PAD) and controlled by Agilent ChemStation software, was used (Agilent, Barcelona, Spain, vers. 6.8). Chromato-156 graphic separations were conducted in a chromatographic column C18 ACE RP18-AR 157 (150 mm × 4.6 mm, 3 μm particle size) protected by a guard col-158 umn ACE 3 C18-AR (7 mm × 13 mm) packed with the same stationary phase (Symta, Madrid, Spain).

All compounds were identified by comparing their retention time and UV/Vis spectrum with analytical reference compounds. Hydroxybenzoic acids and flavan-3-ols were quantified at 280 nm, hydroxycinnamic acids and stilbenes at 320 nm, and flavonols at 360 nm. Phenolic compound quantification was conducted by interpolation of the peak areas into calibration curves of their respective reference compounds’ standard solutions. Samples were analyzed in triplicate.

### 2.6. Analysis of Total Flavan-3-ol Mono-Oligomers and Total Polymers by NP-HPLC

Phenolic compositions were separated based on their polymerization degree and polarity. The same Agilent 1260 chromatographic equipment described above was also used. A 250 mm (172 × 4.6 mm, 5 μm particle size) polar chromatographic column Kromasil 60 DIOL (AzkoNobel, Amsterdam, The Netherlands) was used for the chromatographic separation, equipped also with a precolumn Lichrospher Diol-5 (7 mm × 13 mm). The chromatographic analysis was conducted following the previous method reported by our group, where total polymer procyanidins eluted as a singular peak at the end of the chromatogram, separated from the mono- and oligomeric compounds [8,11]. The analysis was monitored at 280 nm, and a catechin reference curve was used for compound quantification, expressing the results as mg of catechin equivalent (CE)/g extract. Chromatographic analyses were conducted in triplicate.

### 2.7. Total Phenolic Content (TPC)

Total phenolic content was measured according to the well-described protocol of the Folin–Ciocalteu reagent method [29]. Analyses were performed in triplicate, expressing the results as mg of gallic acid equivalents (GAE)/g extract.

### 2.8. Antioxidant Activity

ABTS^•+^ radical scavenging assay was carried out according to the original method described by Re et al. [30]. DPPH^•^ radical scavenging assay was performed by the method described by Brand-Williams et al. [31]. The results obtained through both antioxidant methods were expressed as TEAC value (mmol Trolox/g extract).

ORAC assay was performed following the method reported by our group in previous investigations, expressing the results in TEAC values as µmol Trolox/g sample [28].

All samples were analyzed at least in triplicate for the three described methods.

### 2.9. In Vitro Digestion Process

The digestion process was carried out following the previously performed method, consisting of a static digestion process comprising three digestion steps: oral, stomach, and intestinal steps [8,11]. Briefly, 5 mL of sample (30 mg/mL) was mixed with 0.1 mL of artificial saliva preheated at 37 °C (9.3 mg/mL of amylase from human saliva type XIII-A (Sigma-Aldrich, Madrid, Spain) in CaCl_2_ 1 mM), and mixed for 2 min. Subsequent stomach digestion was performed by adjusting the pH media to 2 with HCl 0.1 M, adding 25 mL of a simulated gastric fluid (127 mg of porcine pepsin from porcine mucosa 536 U/mg, Sigma Aldrich, Madrid, Spain), and stirring for 1 h at 37 °C. After gastric digestion, samples were adjusted to pH 7 with 1 M NaHCO_3_. Then, an intestinal simulated fluid comprising 9.3 mg of pancreatin (Sigma Aldrich, Madrid, Spain) and 115.7 mg of bile salts in 2.8 mL of 10 mM trizma-maleate buffer was added and incubated for 2 h at 37 °C. For stomach and intestinal performance, a titrator Tritinio Plus 877 (Methrom, Herisau, Switzerland) was used, which allowed the pH of the stomach digestion to be maintained at 2 (with HCl 0.1 M) and the intestinal phase to be maintained at pH 7 (1 M NaHCO_3_). After the whole digestion process, the soluble fraction was obtained by filtering the intestinal phase through a 0.45 µm PVDF filter to remove the non-soluble or precipitated compounds, obtaining the denominated soluble fraction.

### 2.10. Caco-2 Cell Transport Experiments

Transepithelial transport assays were conducted following the method described by Nieto et al. [8,11]. Caco-2 cells (American Type Culture Collection, ATCC, Manassas, VA, USA) were maintained in Dulbecco’s Modified Eagle’s Medium (DMEM; Gibco, Barcelona, Spain) supplemented with 10% FBS, 100 U/mL penicillin, 100 mg/mL streptomycin, 1% nonessential amino acids, and 2 mM L-glutamine (Invitrogen, Barcelona, Spain) at 37 °C in at humidified atmosphere containing 5% CO_2_. Prior to cytotoxicity or transepithelial transport assays, the intestinal phase of the digested samples was filtrated through a 0.45 µm PVDF filter to remove insolubilized and precipitated molecules. The filtered and digested cytotoxic was tested in Caco-2 cells using the MTT assay [32], resulting in 100 µL as a suitable volume for transepithelial transport assays. Then, 100 µL were placed in the apical compartment of a Transwell system and incubated for 6 h at 37 °C. Then, the apical and basolateral samples were collected and stored at −20 °C prior to analysis.

### 2.11. Statistical Analyses

Statistical analyses were carried out using the Statgraphics Centurion XVI program (Statpoint Inc., Warrenton, VA, USA). Correlation coefficients between the different experimental data were calculated by using Pearson’s test (*p* ≤ 0.05). Significant differences between the samples were determined through one-way ANOVA analysis, followed by post hoc analyses by the Duncan test for the digestion step analyses (*p* ≤ 0.05).

## 3. Results

### 3.1. Sample Fractionation Through CUF Procedure

The CUF process applied to STE and GSE generated two different fractions of each extract. The molecular fraction of the sample not able to pass through the ultrafiltration membrane *cut-off* remained in the membrane chamber, characterized by high-molecular-weight compounds (≥10 kDa), denominated as retentate. Meanwhile, the fraction of the samples able to pass through the cut-off of the membrane was collected in the centrifuge tube, being constituted by the low molecular compounds’ fraction of the original extracts (<10 kDa), generating the grape stem and seed extract permeates (PSTE and PGSE, respectively). The STE permeate yield was 52% (52 g PSTE/100 g STE), whereas PGSE resulted in a permeate performance of 62% (62 g PGSE/100 g GSE).

**Figure 1:**
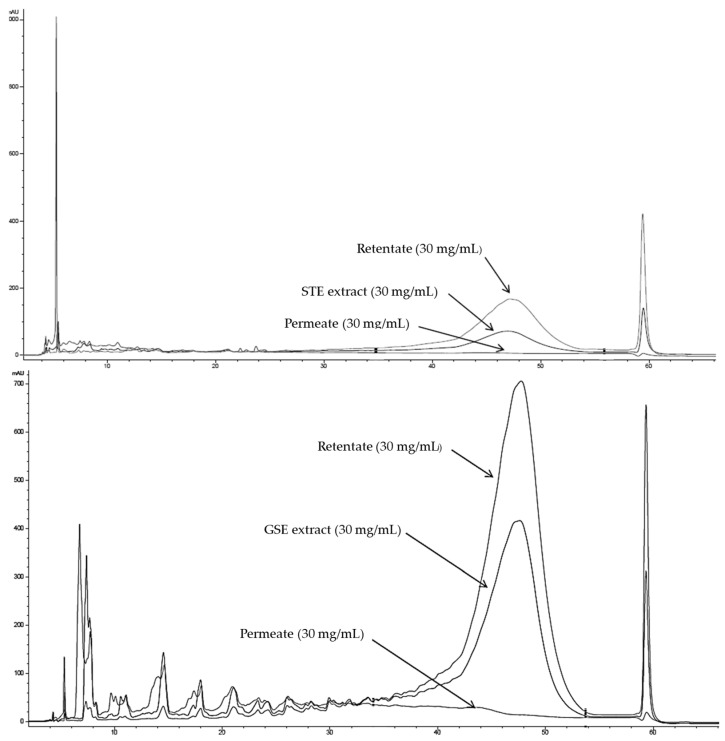
NP-HPLC chromatograms for the original PLE samples, the resulting permeates and retentates (30 mg/mL), for the STE extract (**top**) and GSE extract (**bottom**).

The chromatographic analysis of the polymerization degree (NP-HPLC) confirmed the capability of the CUF fractionation process to separate the high polymeric phenolic compounds from those with lower molecular weight. While a high contribution of polymeric compounds was determined for GSE and STE to total phenolic content (approximately 74% and 65%, respectively), both PSTE and PGSE lacked polymeric compounds and were absolutely composed of mono-oligomeric compounds (Figure 1).

### 3.2. Low-Molecular-Weight Phenolic Composition of PLEs and Permeates

As we have previously reported in the original study, STE and GSE extracts were characterized by an extensive content of procyanidins, with a high content of polymeric proanthocyanidins, mostly in the GSE extract [8,11]. Therefore, both original extracts contained diverse phenolic acids and flavonols, but also stilbenes in the STE extract, whereas in both extracts, falvan-3-ols (monomers, dimmers, and polymers) comprised most of the phenolic constituents (Table 1). Both permeates resulted in a similar phenolic profile trend to the original PLEs used, but the identified mono-oligomeric compounds during RP-HPLC analyses were quantified approximately twice compared with the original extracts (1.7 times for PSTE and 1.6 times for PGSE).

In PSTE, diverse phenolic acids were determined, mainly as gallic acid (1.00 mg/g PSTE), syringic acid (0.416 mg/g PSTE), and caftaric acid (0.256 mg/g PSTE). Additionally, resveratrol (0.464 mg/g PSTE) and diverse quercetin derivatives were identified in the permeate, mainly as quercetin-3-*O*-glucuronide (1.305 mg/g PSTE). Flavan-3-ols were the most abundant compounds in the sample, principally as catechin (4.595 mg/g PSTE), epicatechin (2.653 mg/g PSTE), dimer B1 (1.581 mg/g PSTE), and dimer B2 (0.979 mg/g PSTE).

In PGSE, gallic acid resulted as the most abundant phenolic compound (2.116 mg/g PGSE), and quercetin-3-*O*-glucoside was the main flavonol (0.118 mg/g PGSE), but no stilbenes were identified in the sample. Large amounts of diverse monomeric and dimeric flavan-3-ols were determined. Epicatechin and catechin (68.416 mg/g PGSE and 59.397 mg/g PGSE, respectively) were the principal compounds of the sample, followed by dimer B2 (17.356 mg/g PGSE) and dimer B1 (12.853 mg/g PGSE).

A total of 48% of the STE extract constituents were rejected by the ultrafiltration membrane, allowing to concentrate approximately twice the low-molecular-weight phenolic compounds in PSTE, in concordance with the permeate yield (52%). In general, an enrichment trend of 1.7 times could be observed for these compounds in PSTE compared with STE, except for slightly higher increases for *trans*-resveratrol and procyanidin dimer B2 and lower increments for vanillic acid, 2,3-dihydroxybenzoic acid, ellagic acid, dimer B1, quercetin-3-*O*-rutinoside, and quercetin-3-*O*-glucoside, whereas slight decreases in caftaric acid, quercetin-3-*O*-galactoside, and quercetin-3-*O*-glucuronide were observed in PSTE. In addition, PSTE lacked ethyl gallate. A similar trend was observed for PGSE and GSE. Low-molecular-weight phenolic compounds were increased approximately 1.6 times in PGSE, consistent with the permeate yield (62%), except for slightly lower enrichments in catechin gallate, epicatechin gallate, and quercetin-3-*O*-glucoside.

### 3.3. CUF Effect on TPC

STE and GSE extracts were characterized by high TPC values, being, respectively, 185.33 ± 2.94 mg GAE/g extract and 354.80 ± 3.97 mg GAE/g extract. PSTE and PGSE showed lower TPC values compared to original extracts, being 57.47 ± 1.93 mg GAE/g sample and 210.04 ± 13.03 mg GAE/g sample, respectively.

### 3.4. CUF Effect on Antioxidant Activity

Two different methods were applied to characterize the extracts’ and permeates’ antioxidant capacities, ABTS and ORAC. The antioxidant activity determined by the ABTS method of the STE (3.55 ± 0.21 mmol Trolox/g extract) was significantly lower than that of GSE (9.31 ± 0.23 mmol Trolox/g extract). Similar results were observed for the ORAC method, being 1477.03 ± 172.74 µmol Trolox/g extract and 3135.00 ± 0.40 µmol Trolox/g extract, respectively.

Likewise, the antioxidant activities of the permeates were reduced compared to the original extracts. The antioxidant activities of PSTE were determined as 0.90 ± 0.00 mmol Trolox/g sample and 914.51 ± 60.43 µmol Trolox/g sample, for the ABTS and ORAC methods, respectively. Meanwhile, the TEAC values in PGSE were determined as 4.33 ± 0.02 mmol Trolox/g sample for the ABTS method and 2989.12 ± 85.62 µmol Trolox/g sample for the ORAC method.

### 3.5. CUF Effect on Phenolic Composition During In Vitro Digestion

A similar trend in the phenolic composition of all the samples was observed along the digestion process (Table 2, Table 3, Table 4 and Table 5). Phenolic acids and stilbenes were, in general, more resistant compounds, although important losses in gallic acid occurred during STE and PSTE digestion. On the other hand, slight increases in various phenolic acids could be observed, such as protocatechuic acid or 4-hydroxybenzoic acid, or other minority compounds such as ethyl gallate. Large polymeric procyanidin losses were determined for both GSE and STE extracts, where the oral phase did not significantly influence their content, whereas the gastric phase was the most critical step. During this digestion step, the polymeric phenolic fraction was reduced to 83% and 77%, respectively, reaching a final bioaccessibility close to 20% after the intestinal phase (20% in GSE and 22% in STE).

Extended flavan-3-ol reductions happened regardless of the sample considered. During the STE or PSTE intestinal step, higher flavan-3-ol losses occurred, whereas stomach digestion was shown as the most critical step for GSE and PGSE. Although a similar trend on the phenolic compound reductions can be found between the original PLEs (Table 2 and Table 4) and the generated permeates (Table 3 and Table 5), higher phenolic compounds bioaccessibility values were determined, in general, in the permeates after the whole digestion process, especially in the case of the flavan-3-ols compounds.

Specifically, for STE and PSTE, bioaccessibility was noticeably enhanced in the PSTE digested for gallic acid (73% *vd* 43%), 3-coumaric acid (72% *vd* 0%), resveratrol (86% *vd* 76%), epicatechin (19% *vd* 6%), epicatechin gallate (18% *vd* 0%), and dimer B1 (53% *vd* 16%). Conversely, these enhancements were not observed for catechin (7% *vd* 13%) and for quercetin derivatives, except for quercetin-3-*O*-glucoside.

Therefore, the quantified bioaccessibility for the identified STE mono-oligomers was determined as 29%, compared with 38% for PSTE, resulting in a higher number of mono-oligomer compounds in the permeate digested (5.53 mg/g extract) compared with the original PLE digested (2.45 mg/g extract). Then, many phenolic compounds in the PSTE digested were quantified, in general, twice (or even more) compared to the STE digested, highlighting gallic acid, syringic acid, *trans*-resveratrol, epicatechin, and dimer B1.

Regarding grape seed samples, the bioaccessibility of PGSE compared with GSE was also enhanced, observing these increments principally in flavan-3-ols compounds. Thereby, a noticeably greater amount of catechin (68% *vd* 56%), epicatechin (61% *vd* 38%), and dimer A2 (55% *vd* 17%) was quantified in the PGSE digested. In addition, other kinds of phenolic compounds were also increased in the digested PGSE, which was remarkable in the case of gallic acid, dimer B1, and dimer B2. Hence, larger quantities of identified mono-oligomer phenolic compounds were observed in the digested PGSE (100.22 mg/g extract) compared with the digested GSE (47.58 mg/g extract).

Therefore, the results indicated a trend to enhance the bioaccessibility and the amount of low-molecular-weight phenolic compounds in the permeates digested compared with the original PLEs digested.

### 3.6. CUF Effect on TPC and Antioxidant Activity During In Vitro Digestion

**Figure 2:**
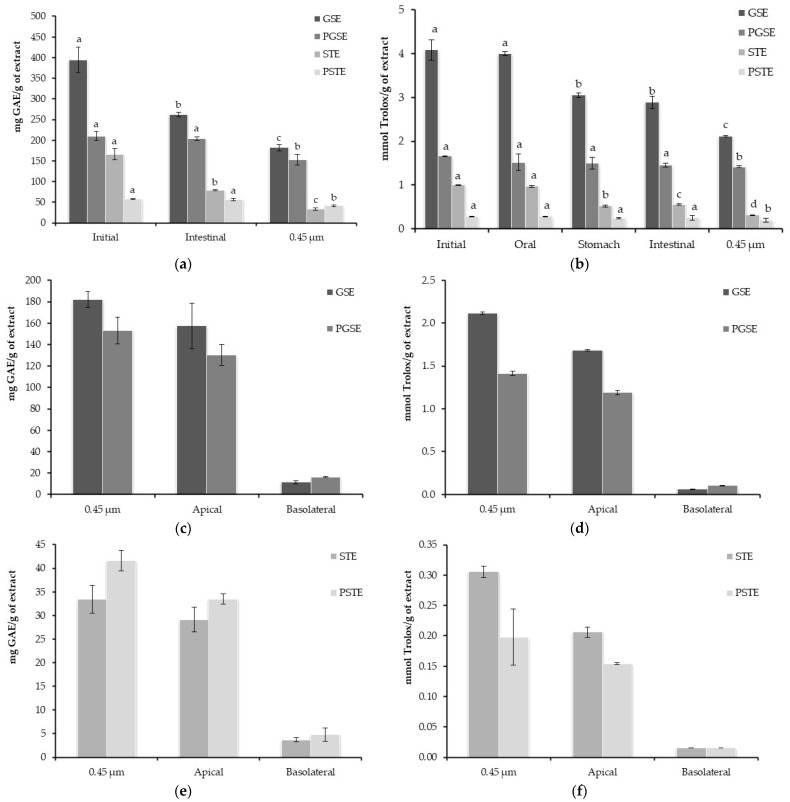
TPC (mg GAE/g extract) and antioxidant activity (TEAC; mmol Trolox/g extract) in GSE, PGSE, STE, and PSTE during the digestion process and transepithelial transport in a Caco-2 cell monolayer absorption process (mean ± S.D.). (**a**) TPC of GSE, PGSE, STE, and PSTE along the digestion process (mg GAE/g extract); (**b**) TEAC values of GSE, PGSE, STE, and PSTE along the digestion process (mmol Trolox/g extract); (**c**) TPC values of GSE and PGSE along transepithelial transport studies in Caco-2 cell monolayers (mg GAE/g extract); (**d**) TEAC values of GSE and PGSE along transepithelial transport studies in Caco-2 cell monolayers (mmol Trolox/g extract); (**e**) TPC values of STE and PSTE along transepithelial transport studies in Caco-2 cell monolayers (mg GAE/g extract); (**f**) TEAC values of STE and PSTE along transepithelial transport studies in Caco-2 cell monolayers (mmol Trolox/g extract). ^a,b,c,d^ Different letters indicate significant statistical differences by Duncan post hoc test (*p* ≤ 0.05).

The digestion process affected the TPC and antioxidant values of both original PLEs (Figure 2). STE reduced the TPC value from 166.04 ± 13.35 mg GAE/g extract in origin to 79.19 ± 1.29 mg GAE/g extract in the digested, showing a bioaccessibility of 48% on TPC. On the other hand, GSE decreased from 394.88 ± 30.54 mg GAE/g to 262.09 ± 6.26 mg GAE/g (66% of bioaccessibility). Conversely, TPC values were not significantly reduced in the permeates. PSTE and PGSE showed TPC values of 57.47 ± 1.58 mg GAE/g and 210.04 ± 10.63 mg GAE/g before the digestion process, respectively, and 55.82 ± 2.72 mg GAE/g and 204.11 ± 4.71 mg GAE/g in the digested samples.

However, all the samples were significantly reduced in TPC values when the samples were submitted to a filtration process through 0.45 µm filters to obtain the soluble fraction. Nevertheless, slight reductions were determined for PSTE and PGSE, while greater losses occurred in STE and GSE samples.

The same trend was observed for the antioxidant activity along the digestion process. GSE and STE reduced the antioxidant activity from 4.09 ± 0.24 and 1.00 ± 0.01 mmol Trolox/g sample, respectively, to 2.89 ± 0.15 and 0.55 ± 0.02 mmol Trolox/g sample. Meanwhile, no significant reductions were determined for PGSE and PSTE, being 1.66 ± 0.00 and 0.28 ± 0.00 mmol Trolox/g sample, respectively, before the digestion process, and 1.46 ± 0.05 and 0.25 ± 0.06 mmol Trolox/g sample after the digestion process. Again, the 0.45 µm applied filtration slightly decreased the antioxidant activity in PGSE and PSTE, whereas important reductions were observed for GSE and STE.

### 3.7. Caco-2 Cell Transport Experiments

A Caco-2 cell monolayer model differentiated to enterocytes was used to study the transepithelial transport of the phenolic compounds that comprised the digested samples. Prior to transport experiments, the digested cytotoxicity was evaluated at 6 h, with 100 µL of the digested extracts (0.45 µm filtrated digested) as the maximum concentration that did not significantly affect cell viability (>99%). These results were confirmed by determining the monolayer cell integrity during exposure experiments by measuring TEER-value. The exposure of the bioaccessible fraction (digested filtrate by 0.45 µm) to 6 h of transport assays allowed us to determine the non-absorbed phenolic fraction (apical) and the absorbed and excreted phenolic fraction, also denominated as a bioavailable fraction (basolateral). TPC, antioxidant activity (DPPH method), and individual mono-oligomeric phenolic composition of both fractions were analyzed.

A similar absorption trend was observed during STE and PSTE transport assays, although some differences could be observed (Table 6). Diverse phenolic compounds were identified in the basolateral fraction of both extracts, mainly composed of phenolic acids such as gallic acid, 4-hydroxybenzoic acid, syringic acid, and caftaric acid, as well as other phenolic compounds such as *trans*-resveratrol, catechin, and quercetin-3-*O*-glucuronide. In general, higher amounts of these phenolic compounds were determined in the PSTE bioavailable fraction compared with STE, except for similar amounts of caftaric acid and catechin, and there were also lower amounts of gallic acid. In addition, noticeably greater amounts of *trans*-resveratrol, as well as a *trans*-resveratrol metabolite, were observed in the PSTE bioavailable fraction. Even more, high quantities of vanillic acid were determined in the PSTE bioavailable fraction, whereas this compound was not determined as bioavailable for STE. Nevertheless, in terms of bioavailability % of the absorbed compounds with respect to the digested samples, similar results were observed for both STE and PSTE.

A similar trend was also determined for the bioavailable fractions of GSE and PGSE (Table 7). Hence, although a similar bioaccessibility % was observed for the identified bioavailable phenolic compounds, the PGSE fraction was characterized by larger amounts of these compounds. Diverse phenolic acids were identified in the basolateral chamber, mainly gallic acid and vanillic acid, while quercetin-3-*O*-glucoside was the only significant bioavailable flavonol, being in similar quantities in both samples. Extended amounts of flavan-3-ols constituted the most abundant phenolics, principally epicatechin and catechin. It is important to note that significant increases in catechin and epicatechin, but mostly dimer B1 and B2, were found in the PGSE basolateral fraction after transepithelial transport assays compared to GSE.

## 4. Discussion

The conventional ultrafiltration process has been extensively reported as a useful method for purifying or concentrating phenolic compounds [25], whereas there is scarce literature on the CAU process. Prodanov et al. [27] investigated the capacity of different pore sizes of CAU membranes (10 kDa, 30 kDa, and 50 kDa) to separate phenolic compounds from almond skin extracts. The authors indicated that the 10 kDa membrane pore size was the most appropriate to separate phenolic compounds by ultrafiltration. Thus, in the present study, a membrane cut-off of 10 kDa was considered suitable to carry out the CUF process to STE and GSE, since it presumably allows a proper retention capacity regarding high molecular phenolic compounds. Podanov et al. [27] used a similar ultrafiltration method, reaching the end point of the process at 1 h through diafiltrations based on three centrifugation steps for 20 min, incorporating new volumes of solvent to remove trapped small molecules. In our study, the cake filtration mechanism did not avoid reaching the dead-end filtration since no liquid sample was observed on the membrane module at the end point of the CUF process (close to 4 h). Although a longer centrifugation time was used, a continuous process without new solvent applications is shown to be an advantage compared to the diafiltration process.

Seed and stem extracts were characterized by high quantities of phenolic compounds and a large amount of polymeric phenolic compounds, especially in the case of grape seed extracts [8,14,28,33]. However, the CUF process yielded a grape seed, and the stem extract permeates lacked a polymeric phenolic fraction. To our knowledge, previous studies have not been conducted with the aim of obtaining low-molecular-weight phenolic fractions from grape stem extracts, despite the method used, whereas grape seed extract ultrafiltration by UV050 membranes with 50 kDa has already been studied, reaching a rejection coefficient of polyphenols close to 80% [25]. In this regard, the filtration yield of PSTE and PGSE (52% and 62%, respectively) corresponded to the removal of the polymeric fraction and, therefore, this value was a consequence of the recovery of most of the mono-oligomeric compounds during the CUF process. This means that in the grape stem permeate (dry), the mono-oligomeric compounds were concentrated close to twice (1.85 times) compared with the original extract, whereas this concentration in PGSE was slightly lower (1.6 times). These results also coincided with the changes in the TPC, being reduced by 68% in PSTE compared with STE and by 41% in PGSE compared with GSE. Accordingly, the antioxidant activity in the permeates was reduced in line with the TPC decreases. A great correlation was observed between the TPC values and the antioxidant activity determined by the ABTS method (r^2^ = 0.98), whereas the worst correlation was determined for the ORAC method (r^2^ = 0.74). Therefore, the decrease in the antioxidant capacity in both PSTE and PGSE was caused by the TPC reduction because of the polymeric compound removal, which was recognized as the main contributor to the antioxidant activity of both PLEs [8,11,28]. In the case of ORAC values, the antioxidant activity is not plentifully governed by the total amount of phenolic compounds, but rather by the sum of the specific activity contribution of each phenolic compound. In this sense, standard or pure phenolic compounds have shown different specific antioxidant activity, even among the diverse antioxidant methods considered [34,35]. Thus, TPC and ORAC values are not always well-fit correlated parameters [36].

When the individual mono-oligomeric composition of the samples is considered, a similar profile trend was determined between the PLEs and their respective permeates. Both the compositions of PSTE and STE coincided with previous research, characterized mostly by flavan-3-ols, mainly as catechin and epicatechin, together with various phenolic acids, such as gallic or caftaric acids, as well as other phenolic molecules, such as resveratrol and diverse quercetin derivatives [14,28,37]. However, a clear increase in the individual mono-oligomer phenolic compound amounts, close to twice (1.85 times), was determined in PSTE compared with STE, agreeing with the previously mentioned concentration effect in low-molecular-weight phenolic compounds in PSTE (also confirmed by RP-HPLC analyses). However, few exceptions were found in this concentration trend. Vanillic acid, ellagic acid, dimer B1, quercetin-3-*O*-rutinoside, and quercetin-3-*O*-glucoside exhibited reduced concentration effects, whereas monogalloyl glucoside, 3-coumaric acid, and epicatechin showed an increased concentration effect.

A similar process was also observed for GSE and PGSE. Hence, both extracts showed the commonly reported phenolic profile for grape seed extracts, generally composed of abundant flavan-3-ols, mainly as epicatechin, catechin, and diverse dimeric compounds, together with various phenolic acids, principally as gallic acid, as well as quercetin derivatives [14,33,38]. Nevertheless, a concentration effect in low-molecular-weight phenolic compounds occurred in PGSE, as was previously mentioned (1.6 times), although catechin, epicatechin, and quercein-3-*O*-glucoside were less concentrated.

Effectiveness of the ultrafiltration processes is governed by diverse factors, such as extract concentration, solvent employed, type and nature of the membrane, membrane pore size, and temperature [24,39,40,41]. Among them, the most influential factor has been identified as the membrane pore size and the phenolic compounds’ interactions with the membrane surface/interior or the membrane–solute–solute interactions [42]. In line with this, specific interactions have been described between phenolic compounds, such as phenolic acids or catechin, and cellulosic materials [43,44]. It is suggested that an increase in (-OH) groups in the phenolic molecules enhances the binding capacity of these compounds with cellulose particles [43], since these interactions have been suggested to occur through absorption onto the hydrophilic surface of cellulose rather than the hydrophobic one, mainly due to Van der Waals’ force and H-bond forces [45]. Therefore, phenolic acids, as well as other phenolic compounds, show specific rejections during ultrafiltration [46,47], where, for example, caftaric acid and protocatechuic acid,are greatly rejected compared to other phenolic acids [47], being in agreement with this study. Therefore, the minimal rejection for some low-molecular-weight phenolics is explained mainly by interactions with the membrane. In addition, the scarce studies conducted with the CUF process to enhance the low-molecular-weight phenolics also show a reduced recovery of some compounds, such as catechin, dimer B1, or B2 [27]. Thus, although most of the low-molecular-weight phenolics are recuperated in the permeate, which is in agreement with this study, CUF effectiveness should be evaluated for each individual phenolic compound during phenolic extract processing. Therefore, further experiments should be conducted that apply the CAU process to other phenolic extracts obtained from food industry by-products.

Nevertheless, the phenolic composition and antioxidant activity of the permeates were not the only parameters modified by the removal of the polymeric phenolic fraction, as bioaccessibility and bioavailability were also affected. Both the original PLEs and the permeates agree with the literature in that no significant changes were observed in the phenolic profile after the oral phase, but both stomach and intestinal digestions were critical steps. Flavan-3-ols and, to a lesser extent flavonols, were the most reduced compounds, whereas stilbenes and phenolic acids resulted as more resistant components [12,14,37]. Slight increases were even observed in several phenolic acids, probably due to the degradation of other phenolic structures, like procyanidins or anthocyanins [48]. It is important to point out that large reductions in polymeric phenolic compounds were quantified for STE and GSE during the digestion process (close to 80%). Previous digestive studies reported great procyanidin losses [28,49,50,51], being a consequence of procyanidin precipitations [13] or degradations [52]. Nevertheless, although a similar trend was observed between PLEs and their permeates, higher total bioaccessibility of identified mono-oligomeric compounds was determined for the permeates, being approximately 38% for PSTE and 60% for PGSE, whereas reduced values were observed in STE and GSE (29% and 46%, respectively). In this regard, enhanced bioaccessibility in PSTE was determined principally for gallic acid, resveratrol, epicatechin, and dimer B1, in addition to other compounds, whereas slight reductions in bioaccessibility % were determined for catechin and quercetin-3-*O*-glucoside. On the other hand, the bioaccessibility of monogalloyl glucoside, catechin, quercetin-3-*O*-glucuronide, and quercetin-3-*O*-glucoside, but mostly 3-coumaric acid, catechin, epicatechin, and dimer A2, resulted in an increased bioaccessibility % in PGSE; meanwhile, the bioaccessibility of caftaric acid, catechin gallate, and dimer B2 was slightly reduced. As a consequence, the total amount of remaining phenolic compounds in the digested PSTE and PGSE increased compared to the digested PLEs. Hence, RP-HPLC analyses revealed that the total identified mono-oligomeric compounds in the digested PSTE were 2.3 times higher compared with PSE, and the digested PGSE showed 2.1 times higher amounts of them compared with GSE.

These results are also in concordance with the TPC and antioxidant values of the samples. STE and GSE were characterized by an intense loss of TPC during the digestion process. Meanwhile, permeate samples maintained the TPC values. These results suggested that the reduction in the TPC values in the original PLEs can be associated with the important reductions in the polymeric fraction during the digestion process. To understand these results, it is important to consider that chromatographic changes in individual phenolic compounds by the digestion process are consequences of phenolic compound modifications [53], where monomers dimerization [54], adduct formations [48], conversions [55], oxidative self-associations [56], and degradations [52], as well as enzyme-bound interactions [57], have been identified. These phenolic modifications clearly affect chromatographic quantification; meanwhile, they could not have an intensive impact on TPC values since the derivatives generated remain in the digestion media, which are able to participate as electron donors, explaining the observed results of this study for RP-HPLC and TPC analyses. Even more, TPC in the permeates only decreased slightly when the soluble fraction was obtained (filtrated digested samples), probably due to a loss of precipitated or enzyme-bound interacted compounds [13,57].

The TPC values also explain the similar trend determined for the antioxidant activity. It should be considered that the whole phenolic composition (that is, TPC values) is related to the antioxidant activity of phenolic extracts during the digestion process [11,58], where the polymeric phenolic fraction represents the major antioxidant activity of winery by-product extracts [33]. Consequently, the antioxidant activity remained in the permeates during the digestion process, whereas this activity was reduced in STE and GSE principally as a consequence of the polymeric fraction reduction [11]. In this regard, a high correlation was found for the polymeric fraction (measured by NP-HPLC) and the antioxidant activity of both STE (r^2^ = 0.999) and GSE (r^2^ = 0.967) extracts during the digestion process, suggesting a great implication of this fraction in the total antioxidant activity.

The results suggest that the phenolic matrix affects the bioaccessibility of the phenolic extracts, with a critical role of the polymeric phenolic fraction. Hence, although the original PLEs were characterized by a greater number of phenolic compounds and therefore, higher antioxidant activity, because of a high loss of polymeric procyanidins in the PLEs, the permeates allowed higher individual mono-oligomeric phenolic compounds, TPC, and antioxidant activity in the digested samples. These results also agree with a previous study conducted by our group, where a PLE grape stem extract with a low amount of polymeric phenolic compounds but with higher amounts of low-molecular-weight phenolic compounds resulted in higher bioaccessibility and antioxidant activity compared with a PLE grape stem extract rich in polymeric procyanidin [11]. However, these results cannot be uniquely explained by a concentration effect on low molecular phenolic compounds derived from the CAU process in the permeates. Total bioaccessibility of PSTE was 2.3 times higher compared with PSE (by NP-HPLC analyses), whereas mono-oligomeric concentration by the CAU process was only 1.85 times higher. The same results were also determined for PGSE, which was concentrated 1.6 times in mono-oligomeric compounds, but resulted in 2.1 times higher amounts of phenolic compounds in the digested samples compared with the digested GSE. Although the effect of the matrix composition has been more studied, this phenolic matrix phenomenon has been, unfortunately, reported less in the literature, and further studies are needed to elucidate the whole action mechanism. In this regard, it is known that at acid pH, interactions between monomers and proanthocyanidins can occur [59]. So, the interaction of mono-oligomers during gastric digestion with further precipitated procyanidins may also contribute to reducing the mono-oligomeric phenolic fraction during the digestion process, although this mechanism needs to be investigated in future research. Thus, considering the results of this study, it can be suggested that removing high molecular phenolics, which are related to precipitation events or interactions with digestive enzymes [52,60], as well as increasing the content in low-molecular-weight phenolic compounds, allows for the enhancement of global phenolic bioaccessibility.

Regarding the specific bioavailability of the low-molecular-weight phenolic compounds, although similar values were determined, quantitative differences were observed between permeated and PLEs. Close bioavailability % were determined for the individual phenolic compounds of both digested PSTE and STE, and even more, lower values were observed for gallic acid, protocatechuic acid, and 4-hydroxybenzoic acid in PSTE digested. Hence, 22% of the compounds of the digested STE were bioavailable, while they were 17% in the digested PSTE. Therefore, both samples resulted in a final bioavailability of the identified mono-oligomeric compounds of 6%, and so, the amount of quantified phenolic compounds in the bioavailable fraction of PSTE was 1.5 times higher than the bioavailable fraction of STE. Therefore, the PSTE bioavailable fraction was noticeably enriched in gallic acid, 4-hydroxybenzoic acid, vanillic acid, syringic acid, *trans*-resveratrol and its metabolite, and quercetin-3-*O*-glucuronide. A similar trend was also observed in grape seed extracts. Digested PGSE and GSE resulted in similar bioaccessibility % of the individual phenolic compounds, but increases were determined for caftaric acid, epicatechin gallate, dimer B2, and dimer A2 in digested PGSE. Meanwhile, it was reduced for catechin and epicatechin. Then, 16% of the mono-oligomeric compounds of the digested GSE were bioavailable, comprising a total bioavailability of the extract of 7.5%. Conversely, 10% of the digested PGSE was bioavailable, resulting in a total bioavailability of the extracts of 6%. Nevertheless, it must not be forgotten that beyond the lower bioavailability observed for the permeate, their bioavailable fraction comprised greater amounts of these individual mono-oligomeric phenolic compounds, even those characterized by a lower bioavailability % (close to 1.5 times greater than the GSE). Hence, gallic acid, vanillic acid, and especially the identified fralvan-3-ols compounds were more abundant in the bioavailable PGSE fraction.

Indeed, greater results were determined when the TPC values were considered, which also comprised non-identified mono-oligomeric compounds during HPL-RP analyses. In this regard, the bioavailability of TPC was 12% and 11% for PSTE and PGSE digested, respectively, and 11% and 6% for the digested STE and GSE. Therefore, PSTE and PGSE resulted in total bioavailability of 8%, whereas STE and GSE were only 2 and 3% bioavailable. These results, based on the higher amount of TPC, as well as higher quantifications of individual mono-oligomeric phenolic compounds in the permeated bioavailable fraction, also explain their greater antioxidant activity compared with the STE and GSE bioavailable fraction.

This augmented abundance of mono-oligomeric phenolic compounds in the bioavailable fraction of the permeates was probably due to their enhanced bioaccessibility, allowing them to absorb a larger number of these compounds. As we mentioned before, the improved bioaccessibility is not explained only by the increment of mono-oligomeric compounds in the sample but also by the absence of polymeric compounds during the digestion process. It is known that high-molecular-weight phenolics, such as procyanidins, are not bioavailable compounds [49,50,51], whereas low-molecular-weight phenolics show a wide intestinal absorption [11,28,61,62]. In addition, during the digestion process, the phenolic matrix can condition the phenolic compounds’ bioaccessibility or bioavailability. In this context, previous research conducted by our group indicated that the amount of polymeric phenolic compounds in grape stem extracts can affect and reduce the bioaccessibility of these compounds along the digestion process and, consequently, impact the final bioavailability [11]. Consequently, the final bioavailability of the extracts is also affected by the phenolic matrix. This is even more so the case if it is considered that phenolic compounds absorption is mediated by different biological mechanisms, such as paracellular absorption [57,61], transport through monocarboxylic acid transporter (MCT) [61], saturable protein transporters [63], or enterocytes metabolism [62]. In this regard, greater *trans*-resveratrol amounts were absorbed in PSTE compared to STE, resulting not only in increased quantities of a resveratrol metabolite but also noticeable enhancements of the native *trans*-resveratrol compound due to a saturation transport effect derived from the enhanced bioaccessibility of this compound in PSTE [62,63]. The results of this study are also in concordance with in vivo studies, where rats fed with a red wine extract showed higher amounts of flavan-3-ol monomers in blood samples than those fed with a grape seed extract with a higher phenolic content, suggesting a lower bioavailability of the grape seed extract as a consequence of its higher polymeric proanthocyanidin content [51]. Then, as was previously pointed out in the literature, it is not always the case that the most plentiful matrix compounds are the ones to give way to greater results [64].

## 5. Conclusions

The CUF process using membranes with a pore size of 10 kDa allowed the separation of high- and low-molecular-weight phenolic fractions. As a result, the permeates from the grape stem and seed extracts were generated with lower TPC and antioxidant activity due to the removal of polymeric procyanidins, while low-molecular-weight phenolics were increased close to twofold. The removal of the polymeric fraction was critical in allowing the permeates to reach higher bioaccessibility values after the digestion process. Consequently, higher phenolic total content, individual mono-oligomeric phenolic compound amount, and antioxidant activity were achieved in the permeates’ bioavailable fractions. Therefore, the results of this study suggest that the global phenolic composition of the extract substantially affects the bioaccessibility and bioavailability of the phenolic compounds. Specifically, removing high polymeric compounds, as well as enriching the extract in low-molecular-weight phenolic compounds, seems to allow greater amounts of remaining compounds after the digestion, and then increases the total phenolic compounds absorbed in the intestine. In this context, the application of the CUF process with an appropriate membrane *cut-off* was highlighted as a suitable method for producing permeates that lack polymeric procyanidins and are enriched with low-molecular-weight phenolic compounds, and therefore, increasing the amount of absorbed phenolic compounds. Then, removal of the polymeric fraction of extracts obtained from food industry by-products could trigger more efficient bioactive extracts, as well as obtain a high polymeric phenolic extract for industrial uses, each fraction thus being more efficient in its purposes. However, further analyses must be conducted using CUF methodology on other phenolic matrices, as well as analyses in preclinical in vivo studies to evaluate the impact of polymeric compound removal and mono-oligomers’ concentration on the extract absorption and functionality.

## Figures and Tables

**Table 1:** Individual low-molecular-weight phenolic compound composition determined by RP-HPLC analyses in the original PLE grape stem extract (STE) and grape seed extract (GSE), as well as in the resulting permeates (PSTE and PGSE, respectively), expressing the results as mg/g extract (mean ± S.D.) (*n* = 3).

	STE	PSTE	GSE	PGSE
**Hydroxybenzoic acids**				
Gallic acid	0.622 ± 0.003	1.000 ± 0.044	1.355 ± 0.004	2.116 ± 0.053
Protocatechuic acid	0.010 ± 0.000	0.020 ± 0.001	0.008 ± 0.000	0.011 ± 0.000
Monogalloyl glucoside	0.012 ± 0.001	0.043 ± 0.003	0.524 ± 0.001	0.845 ± 0.042
4-Hydroxybenzoic acid	0.058 ± 0.003	0.107 ± 0.002	<LoD	<LoD
Vanillic acid	0.209 ± 0.013	0.235 ± 0.047	<LoD	<LoD
Syringic acid	0.277 ± 0.004	0.416 ± 0.014	<LoD	<LoD
**Hydroxycinnamic acids**				
Caftaric acid	0.165 ± 0.002	0.256 ± 0.011	0.029 ± 0.000	0.045 ± 0.004
3-Coumaric acid	0.003 ± 0.000	0.008 ± 0.001	<LoD	0.028 ± 0.007
**Derived from phenolic acids**				
Ethyl gallate	0.010 ± 0.001	0.021 ± 0.001	<LoD	<LoD
Ellagic acid	0.077 ± 0.004	0.078 ± 0.001	0.016 ± 0.002	Nd
**Stilbenes**				
*trans*-Piceid	0.014 ± 0.000	0.027 ± 0.000	<LoD	<LoD
*trans*-Resveratrol	0.263 ± 0.004	0.464 ± 0.020	<LoD	<LoD
**Flavan-3-ols**				
Catechin	2.749 ± 0.010	4.595 ± 0.238	32.405 ± 0.755	59.397 ± 2.487
Epicatechin	0.851 ± 0.003	2.653 ± 0.145	44.217 ± 0.089	68.416 ± 2.557
Catechin gallate			0.034 ± 0.004	0.046 ± 0.007
Epicatechin gallate	0.230 ± 0.003	0.427 ± 0.041	3.927 ± 0.019	5.388 ± 0.457
Dimer B1	1.562 ± 0.004	1.581 ± 0.056	7.761 ± 0.017	12.853 ± 1.158
Dimer B2	0.508 ± 0.027	0.979 ± 0.048	11.856 ± 0.015	17.356 ± 1.657
Dimer A2	<LoD	<LoD	0.440 ± 0.018	0.613 ± 0.012
**Flavonols**				
Quercetin-3-*O*-galactoside	0.018 ± 0.001	0.032 ± 0.002	<LoD	<LoD
Quercetin-3-*O*-rutinoside	0.019 ± 0.001	0.027 ± 0.003	<LoD	<LoD
Quercetin-3-*O*-glucuronide	0.784 ± 0.010	1.305 ± 0.077	0.013 ± 0.000	0.026 ± 0.011
Quercetin-3-*O*-glucoside	0.106 ± 0.004	0.107 ± 0.005	0.101 ± 0.000	0.118 ± 0.005
Quercetin	0.004 ± 0.000	0.019 ± 0.001	<LoD	<LoD
**Σ Phenolic compounds**	8.56 ± 0.098	14.41 ± 0.764	102.73 ± 0.925	167.27 ± 8.457

LoD = Limit of detection.

**Table 2:** Individual low-molecular-weight phenolic compounds’ composition determined by RP-HPLC analyses in the STE along the digestion process, with the results expressed as mg/g extract (mean ± S.D.) (*n* = 3).

	Initial	Oral	Stomach	Intestinal
Gallic acid	0.622 ± 0.030 ^b^	0.645 ± 0.001 ^a^	0.504 ± 0.002 ^c^	0.270 ± 0.024 ^d^
Protocatechuic acid	0.010 ± 0.000 ^a^	0.011 ± 0.000 ^a^	0.010 ± 0.000 ^a^	0.011 ± 0.002 ^a^
Monogalloyl glucoside	0.012 ± 0.001 ^a^	0.010 ± 0.000 ^b^	<LoD	<LoD
4-Hydroxybenzoic acid	0.058 ± 0.003 ^c^	0.060 ± 0.002 ^c^	0.068 ± 0.002 ^b^	0.074 ± 0.004 ^a^
Vanillic acid	0.209 ± 0.013 ^a^	0.201 ± 0.019 ^a^	Co	Co
Syringic acid	0.277 ± 0.004 ^b^	0.261 ± 0.012 ^c^	0.291 ± 0.002 ^a^	0.295 ± 0.008 ^a^
Ethyl gallate	0.010 ± 0.001 ^c^	0.010 ± 0.002 ^c^	0.014 ± 0.000 ^b^	0.021 ± 0.001 ^a^
Ellagic acid	0.077 ± 0.004 ^a^	0.072 ± 0.002 ^b^	0.037 ± 0.001 ^d^	0.046 ± 0.001 ^c^
Caftaric acid	0.165 ± 0.002 ^b^	0.173 ± 0.000 ^a^	0.148 ± 0.001 ^c^	0.130 ± 0.005 ^d^
3-Coumaric acid	0.003 ± 0.000 ^a^	0.003 ± 0.000 ^a^	<LoD	<LoD
*trans*-Piceid	0.014 ± 0.000 ^a^	0.015 ± 0.000 ^a^	0.014 ± 0.000 ^a^	0.014 ± 0.000 ^a^
*trans*-Resveratrol	0.263 ± 0.004 ^a^	0.267 ± 0.001 ^a^	0.218 ± 0.001 ^b^	0.199 ± 0.015 ^c^
Catechin	2.749 ± 0.010 ^a^	2.755 ± 0.169 ^a^	1.885 ± 0.002 ^b^	0.368 ± 0.037 ^c^
Epicatechin	0.851 ± 0.003 ^a^	0.892 ± 0.023 ^a^	0.683 ± 0.001 ^b^	0.052 ± 0.029 ^c^
Epicatechin gallate	0.230 ± 0.003 ^a^	0.234 ± 0.004 ^a^	<LoD	<LoD
Dimer B1	1.562 ± 0.004 ^a^	1.577 ± 0.055 ^a^	1.434 ± 0.004 ^b^	0.248 ± 0.044 ^c^
Dimer B2	0.508 ± 0.027 ^a^	0.521 ± 0.010 ^a^	0.293 ± 0.007 ^b^	Co
Quercetin-3-*O*-galactoside	0.018 ± 0.001 ^a^	0.018 ± 0.001 ^a^	0.005 ± 0.001 ^c^	0.009 ± 0.003 ^b^
Quercetin-3-*O*-rutinoside	0.019 ± 0.001 ^a^	0.018 ± 0.001 ^a^	0.015 ± 0.001 ^b^	0.014 ± 0.002 ^b^
Quercetin-3-*O*-glucuronide	0.784 ± 0.010 ^a^	0.797 ± 0.006 ^a^	0.555 ± 0.002 ^c^	0.629 ± 0.051 ^b^
Quercetin-3-*O*-glucoside	0.106 ± 0.004 ^a^	0.110 ± 0.004 ^a^	0.082 ± 0.002 ^b^	0.073 ± 0.003 ^c^
Quercetin	0.004 ± 0.000 ^a^	0.005 ± 0.000 ^a^	0.001 ± 0.000 ^b^	<LoD

^a,b,c,d^ Letters indicate significant differences in compound content between digestion steps by Duncan test (*p* < 0.05). Co = coelute. LoD = limit of detection.

**Table 3:** Individual low-molecular-weight phenolic compounds’ composition determined by RP-HPLC analyses in the PSTE along the digestion process, with the results expressed as mg/g extract (mean ± S.D.) (*n* = 3).

	Initial	Oral	Stomach	Intestinal
Gallic acid	1.000 ± 0.044 ^a^	1.092 ± 0.056 ^a^	0.716 ± 0.055 ^b^	0.729 ± 0.009 ^b^
Protocatechuic acid	0.020 ± 0.001 ^a^	0.021 ± 0.002 ^a^	0.019 ± 0.001 ^a^	0.017 ± 0.000 ^b^
Monogalloyl glucoside	0.043 ± 0.003 ^a^	0.046 ± 0.004 ^a^	0.022 ± 0.001 ^b^	<LoD
4-Hydroxybenzoic acid	0.107 ± 0.002 ^c^	0.113 ± 0.006 ^c^	0.126 ± 0.006 ^b^	0.162 ± 0.015 ^a^
Vanillic acid	0.235 ± 0.047 ^a^	0.229 ± 0.014 ^a^	0.238 ± 0.033 ^a^	0.258 ± 0.054 ^a^
Syringic acid	0.416 ± 0.014 ^b^	0.459 ± 0.027 ^a,b^	0.469 ± 0.015 ^a^	0.437 ± 0.012 ^a,b^
Ethyl gallate	0.021 ± 0.001 ^c^	0.023 ± 0.001 ^c^	0.029 ± 0.005 ^b^	0.050 ± 0.008 ^a^
Ellagic acid	0.078 ± 0.001 ^a^	0.083 ± 0.005 ^a^	0.013 ± 0.000 ^c^	0.020 ± 0.001 ^b^
Caftaric acid	0.256 ± 0.011 ^a^	0.281 ± 0.018 ^a^	0.188 ± 0.017 ^b^	0.132 ± 0.006 ^c^
3-Coumaric acid	0.008 ± 0.001 ^a,b^	0.010 ± 0.001 ^a^	0.005 ± 0.001 ^c^	0.006 ± 0.001 ^a,b^
*trans*-Piceid	0.027 ± 0.000 ^a^	0.028 ± 0.000 ^a^	0.028 ± 0.002 ^a^	0.028 ± 0.000 ^a^
*trans*-Resveratrol	0.464 ± 0.020 ^b^	0.507 ± 0.029 ^a^	0.461 ± 0.011 ^b^	0.396 ± 0.001 ^c^
Catechin	4.595 ± 0.238 ^a^	5.003 ± 0.340 ^a^	2.165 ± 0.078 ^b^	0.329 ± 0.042 ^c^
Epicatechin	2.653 ± 0.145 ^a^	2.800 ± 0.222 ^a^	0.992 ± 0.096 ^b^	0.515 ± 0.060 ^c^
Epicatechin gallate	0.427 ± 0.041 ^a^	0.462 ± 0.027 ^a^	0.110 ± 0.008 ^b^	0.076 ± 0.006 ^c^
Dimer B1	1.581 ± 0.056 ^a^	1.740 ± 0.109 ^a^	1.250 ± 0.003 ^b^	0.831 ± 0.052 ^c^
Dimer B2	0.979 ± 0.048 ^a^	0.971 ± 0.030 ^a^	0.827 ± 0.021 ^b^	0.625 ± 0.085 ^c^
Quercetin-3-*O*-galactoside	0.032 ± 0.002 ^a^	0.036 ± 0.003 ^a^	0.018 ± 0.004 ^b^	0.013 ± 0.003 ^c^
Quercetin-3-*O*-rutinoside	0.027 ± 0.003 ^a^	0.031 ± 0.002 ^a^	0.024 ± 0.002 ^b^	0.016 ± 0.002 ^c^
Quercetin-3-*O*-glucuronide	1.305 ± 0.077 ^a^	1.430 ± 0.092 ^a^	1.005 ± 0.041 ^b^	0.787 ± 0.068 ^c^
Quercetin-3-*O*-glucoside	0.107 ± 0.005 ^b^	0.120 ± 0.006 ^a^	0.082 ± 0.004 ^c^	0.091 ± 0.024 ^b,c^
Quercetin	0.019 ± 0.000 ^a^	0.020 ± 0.001 ^a^	<LoD	<LoD

^a,b,c^ Letters indicate significant differences in compound content between digestion steps by Duncan test (*p* < 0.05). LoD = limit of detection.

**Table 4:** Individual low-molecular-weight phenolic compounds’ composition determined by RP-HPLC analyses in the GSE along the digestion process, with the results expressed as mg/g extract (mean ± S.D.) (*n* = 3).

	Initial	Oral	Stomach	Intestinal
Gallic acid	1.355 ± 0.004 ^a^	1.267 ± 0.008 ^b^	1.278 ± 0.011 ^b^	1.214 ± 0.009 ^c^
Protocatechuic acid	0.008 ± 0.000 ^b^	0.007 ± 0.000 ^b^	0.008 ± 0.001 ^b^	0.010 ± 0.000 ^a^
Monogalloyl glucoside	0.524 ± 0.001 ^a^	0.532 ± 0.007 ^a^	0.390 ± 0.003 ^b^	0.382 ± 0.032 ^b^
Ellagic acid	0.016 ± 0.002 ^a^	0.015 ± 0.000 ^a,b^	0.013 ± 0.001 ^b,c^	0.012 ± 0.000 ^c^
Caftaric acid	0.029 ± 0.000 ^a^	0.025 ± 0.001 ^b^	0.023 ± 0.000 ^c^	0.021 ± 0.001 ^d^
3-Coumaric acid	0.025 ± 0.000 ^a^	0.025 ± 0.001 ^a^	0.014 ± 0.000 ^b^	0.005 ± 0.000 ^c^
Catechin	32.405 ± 0.755 ^a^	31.643 ± 0.049 ^a^	21.349 ± 0.496 ^b^	18.028 ± 0.226 ^c^
Epicatechin	44.217 ± 0.089 ^a^	41.786 ± 0.737 ^b^	22.143 ± 0.044 ^c^	16.782 ± 0.106 ^d^
Catechin gallate	0.034 ± 0.004 ^a^	0.036 ± 0.002 ^a^	0.008 ± 0.001 ^c^	0.013 ± 0.001 ^b^
Epicatechin gallate	3.927 ± 0.019 ^a^	3.427 ± 0.031 ^b^	1.510 ± 0.003 ^d^	1.588 ± 0.002 ^c^
Dimer B1	7.761 ± 0.017 ^b^	7.951 ± 0.132 ^a^	7.730 ± 0.021 ^b^	2.874 ± 0.077 ^c^
Dimer B2	11.856 ± 0.015 ^a^	11.149 ± 0.205 ^b^	6.848 ± 0.008 ^c^	6.470 ± 0.082 ^d^
Dimer A2	0.440 ± 0.018 ^a^	0.473 ± 0.021 ^a^	0.239 ± 0.006 ^b^	0.075 ± 0.028 ^c^
Quercetin-3-*O*-glucuronide	0.013 ± 0.000 ^a^	0.010 ± 0.000 ^b^	0.002 ± 0.001 ^c^	0.010 ± 0.001 ^b^
Quercetin-3-*O*-glucoside	0.101 ± 0.000 ^a^	0.094 ± 0.001 ^a^	0.063 ± 0.006 ^b^	0.067 ± 0.001 ^b^
Quercetin	<LoD	<LoD	<LoD	<LoD

^a,b,c,d^ Letters indicate significant differences in compound content between digestion steps by Duncan test (*p* < 0.05). LoD = limit of detection.

**Table 5:** Individual low-molecular-weight phenolic compounds’ composition determined by RP-HPLC analyses in the PGSE along the digestion process, with the results expressed as mg/g extract (mean ± S.D.) (*n* = 3).

	Initial	Oral	Stomach	Intestinal
Gallic acid	2.116 ± 0.053 ^a^	2.021 ± 0.000 ^a^	2.144 ± 0.012 ^a^	1.826 ± 0.171 ^b^
Protocatechuic acid	0.0011 ± 0.000 ^b^	0.009 ± 0.000 ^c^	0.013 ± 0.000 ^a^	0.014 ± 0.001 ^a^
Monogalloyl glucoside	0.845 ± 0.042 ^a^	0.801 ± 0.001 ^a,b^	0.762 ± 0.042 ^b^	0.777 ± 0.054 ^a,b^
Ellagic acid	<LoD	<LoD	<LoD	<LoD
Caftaric acid	0.045 ± 0.004 ^a^	0.048 ± 0.000 ^b^	0.039 ± 0.001 ^b^	0.028 ± 0.001 ^c^
3-Coumaric acid	0.028 ± 0.007 ^a^	0.032 ± 0.001 ^a^	0.025 ± 0.002 ^a,b^	0.018 ± 0.003 ^b^
Catechin	59.397 ± 2.847 ^a^	54.818 ± 0.573 ^b^	40.689 ± 0.427 ^c^	40.421 ± 1.500 ^c^
Epicatechin	68.416 ± 2.557 ^a^	59.386 ± 0.187 ^b^	47.904 ± 0.587 ^c^	41.712 ± 1.397 ^d^
Catechin gallate	0.046 ± 0.007 ^a^	0.049 ± 0.001 ^a^	0.014 ± 0.002 ^b^	0.006 ± 0.000 ^c^
Epicatechin gallate	5.388 ± 0.457 ^a^	4.540 ± 0.003 ^b^	2.620 ± 0.185 ^c^	1.831 ± 0.137 ^d^
Dimer B1	12.853 ± 1.158 ^a^	13.651 ± 0.160 ^a^	8.968 ± 0.019 ^b^	5.637 ± 0.201 ^c^
Dimer B2	17.356 ± 1.657 ^a^	15.578 ± 0.007 ^b^	11.353 ± 0.026 ^c^	7.501 ± 0.264 ^d^
Dimer A2	0.613 ± 0.012 ^a^	0.652 ± 0.011 ^b^	0.311 ± 0.002 ^c^	0.335 ± 0.034 ^d^
Quercetin-3-*O*-glucuronide	0.026 ± 0.011 ^a^	0.020 ± 0.001 ^a^	0.016 ± 0.002 ^a^	0.023 ± 0.003 ^a^
Quercetin-3-*O*-glucoside	0.118 ± 0.005 ^a^	0.121 ± 0.012 ^a^	0.108 ± 0.004 ^a^	0.090 ± 0.003 ^b^
Quercetin	<LoD	<LoD	<LoD	<LoD

^a,b,c,d^ Letters indicate significant differences in a compound content between digestion steps by Duncan test (*p* < 0.05). LoD = limit of detection.

**Table 6:** Individual low-molecular-weight phenolic compounds’ composition determined by RP-HPLC analyses in the STE and PSTE along the transepithelial transport in Caco-2 cell monolayers, with the results expressed as mg/g extract (mean ± S.D.) (*n* = 3).

	STE			PSTE		
	0.45 µm	Apical	Basolateral	0.45 µm	Apical	Basolateral
Gallic acid	0.270 ± 0.024 ^a^	0.217 ± 0.002 ^b^	0.064 ± 0.008 ^c^	0.729 ± 0.009 ^a^	0.258 ± 0.009 ^b^	0.026 ± 0.002 ^c^
Protocatechuic acid	0.011 ± 0.002 ^a^	0.008 ± 0.000 ^b^	0.002 ± 0.000 ^c^	0.017 ± 0.000 ^a^	0.014 ± 0.000 ^b^	0.002 ± 0.000 ^c^
Monogalloyl glucoside	<LoD	<LoD	<LoD	<LoD	<LoD	<LoD
4-Hydroxybenzoic acid	0.074 ± 0.008 ^a^	0.049 ± 0.006 ^b^	0.028 ± 0.002 ^c^	0.162 ± 0.015 ^a^	0.132 ± 0.005 ^b^	0.040 ± 0.002 ^c^
Vanillic acid	Co	<LoD	<LoD	0.258 ± 0.054 ^a^	Co	0.201 ± 0.005 ^b^
Syringic acid	0.295 ± 0.23 ^a^	0.212 ± 0.18 ^b^	0.076 ± 0.05 ^c^	0.437 ± 0.012 ^a^	0.271 ± 0.004 ^b^	0.102 ± 0.008 ^c^
Ethyl gallate	0.021 ± 0.000 ^a^	0.009 ± 0.002 ^b^	<LoD	0.050 ± 0.008 ^a^	0.012 ± 0.003 ^b^	<LoD
Ellagic acid	0.046 ± 0.001 ^a^	0.032 ± 0.002 ^b^	<LoD	0.020 ± 0.001 ^a^	0.013 ± 0.001 ^b^	<LoD
Caftaric acid	0.130 ± 0.005 ^a^	0.071 ± 0.003 ^b^	0.017 ± 0.001 ^c^	0.132 ± 0.006 ^a^	0.064 ± 0.004 ^b^	0.015 ± 0.001 ^c^
3-Coumaric acid	<LoD	<LoD	<LoD	0.006 ± 0.001	<LoD	<LoD
*trans*-Piceid	0.014 ± 0.000 ^a^	0.013 ± 0.000 ^b^	0.003 ± 0.000 ^c^	0.028 ± 0.000 ^a^	0.018 ± 0.000 ^c^	0.003 ± 0.000 ^b^
*trans*-Resveratrol	0.199 ± 0.015 ^a^	0.006 ± 0.000 ^c^	0.019 ± 0.000 ^b^	0.396 ± 0.001 ^a^	0.009 ± 0.000 ^c^	0.038 ± 0.001 ^b^
Metabolite of resveratrol	<LoD	0.139 ± 0.001 ^a^	0.070 ± 0.001 ^b^	<LoD	0.158 ± 0.002 ^a^	0.149 ± 0.005 ^a^
Catechin	0.368 ± 0.037 ^a^	Co	0.215 ± 0.001 ^b^	0.329 ± 0.042 ^a^	Co	0.202 ± 0.009 ^b^
Epicatechin	0.052 ± 0.029	<LoD	<LoD	0.515 ± 0.060 ^a^	0.141 ± 0.033 ^b^	<LoQ
Epicatechin gallate	<LoD	<LoD	<LoD	0.076 ± 0.006	<LoD	<LoD
Dimer B1	0.248 ± 0.044 ^a^	0.113 ± 0.008 ^b^	<LoD	0.166 ± 0.052	<LoD	<LoD
Dimer B2	Co	<LoD	<LoD	0.625 ± 0.085	<LoQ	<LoD
Quercetin-3-*O*-galactoside	0.009 ± 0.003 ^a^	0.009 ± 0.000 ^a^	<LoD	0.013 ± 0.003 ^a^	0.005 ± 0.001 ^b^	<LoD
Quercetin-3-*O*-rutinoside	0.014 ± 0.002 ^a^	0.013 ± 0.000 ^a^	<LoD	0.016 ± 0.002	<LoQ	<LoD
Quercetin-3-*O*-glucuronide	0.629 ± 0.051 ^a^	0.507 ± 0.004 ^b^	0.040 ± 0.001 ^c^	0.787 ± 0.077 ^a^	0.391 ± 0.010 ^b^	0.03 ± 0.001 ^c^
Quercetin-3-*O*-glucoside	0.073 ± 0.003 ^a^	0.038 ± 0.000 ^b^	<LoQ	0.091 ± 0.024 ^a^	0.075 ± 0.003 ^b^	<LoQ
Quercetin	<LoD	<LoD	<LoD	<LoQ	<LoD	<LoD

^a,b,c^ Letters indicate significant differences in compound content between digested, apical, and basolateral fractions by Duncan test (*p* < 0.05). Co = coelute. LoD = Limit of detection. LoQ = Limit of quantification.

**Table 7:** Individual low-molecular-weight phenolic compound composition determined by RP-HPLC analyses in the GSE and PGSE along the transepithelial transport in Caco-2 cell monolayers, with the results expressed as mg/g extract (mean ± S.D.) (*n* = 3).

	GSE			PGSE		
	0.45 µm	Apical	Basolateral	0.45 µm	Apical	Basolateral
Gallic acid	1.213 ± 0.009 ^a^	0.845 ± 0.004 ^b^	0.052 ± 0.003 ^c^	1.826 ± 0.171 ^a^	1.206 ± 0.003 ^b^	0.085 ± 0.001 ^c^
Protocatechuic acid	0.010 ± 0.000 ^b^	0.018 ± 0.000 ^a^	0.002 ± 0.000 ^c^	0.014 ± 0.001 ^a^	Co	0.002 ± 0.000 ^b^
Monogalloyl glucoside	0.382 ± 0.032 ^a^	0.312 ± 0.021 ^b^	<LoQ	0.777 ± 0.054 ^a^	0.673 ± 0.035 ^b^	0.058 ± 0.014 ^c^
Vanillic acid	<LoD	<LoD	0.072 ± 0.001 ^a^	<LoD	<LoD	0.118 ± 0.006 ^a^
Ellagic acid	0.017 ± 0.000 ^a^	0.007 ± 0.000 ^b^	<LoD	<LoD	<LoD	<LoD
Caftaric acid	0.021 ± 0.001 ^a^	0.021 ± 0.000 ^b^	<LoD	0.028 ± 0.001 ^a^	Co	0.012 ± 0.000 ^b^
3-Coumaric acid	0.005 ± 0.000 ^b^	0.018 ± 0.001 ^a^	<LoD	0.018 ± 0.003 ^a^	0.018 ± 0.001 ^a^	<LoQ
Catechin	18.03 ± 0.227 ^a^	13.16 ± 0.383 ^b^	3.315 ± 0.071 ^c^	40.42 ± 1.500 ^a^	25.28 ± 0.280 ^b^	3.98 ± 0.027 ^c^
Epicatechin	16.78 ± 0.107 ^a^	13.67 ± 1.037 ^b^	3.671 ± 0.087 ^c^	41.71 ± 1.397 ^a^	29.48 ± 0.280 ^b^	4.223 ± 0.040 ^c^
Catechin gallate	0.013 ± 0.003 ^a^	0.004 ± 0.001 ^b^	0.001 ± 0.000 ^c^	0.006 ± 0.000 ^a^	0.006 ± 0.001 ^a^	0.001 ± 0.000 ^b^
Epicatechin gallate	1.589 ± 0.002 ^a^	0.704 ± 0.081 ^b^	0.191 ± 0.001 ^c^	1.831 ± 0.137 ^a^	0.940 ± 0.008 ^b^	0.291 ± 0.004 ^c^
Dimer B1	2.874 ± 0.077 ^b^	3.013 ± 0.029 ^a^	0.080 ± 0.003 ^c^	5.637 ± 0.201 ^a^	4.448 ± 0.030 ^b^	0.465 ± 0.005 ^c^
Dimer B2	6.470 ± 0.083 ^a^	5.849 ± 0.073 ^b^	0.253 ± 0.002 ^c^	7.501 ± 0.264 ^b^	8.418 ± 0.150 ^a^	1.034 ± 0.025 ^c^
Dimer A2	0.075 ± 0.028 ^a^	0.081 ± 0.007 ^b^	<LoD	0.335 ± 0.034 ^a^	0.195 ± 0.003 ^b^	0.042 ± 0.002 ^c^
Quercetin-3-*O*-glucuronide	0.010 ± 0.001 ^b^	0.012 ± 0.001 ^a^	<LoD	0.023 ± 0.003 ^a^	0.026 ± 0.001 ^a^	<LoQ
Quercetin-3-*O*-glucoside	0.067 ± 0.001 ^a^	0.039 ± 0.002 ^b^	0.023 ± 0.000 ^c^	0.090 ± 0.003 ^a^	0.066 ± 0.001 ^b^	0.030 ± 0.000 ^c^
Quercetin	<LoD	<LoD	<LoD	<LoQ	<LoD	<LoD

^a,b,c^ Letters indicate significant differences in compound content between digested, apical, and basolateral fractions by Duncan test (*p* < 0.05). Co = coelute. LoD = Limit of detection. LoQ = Limit of quantification.

## Data Availability

The original contributions presented in this study are included in the article. Further inquiries can be directed to the corresponding author.

## Abbreviations

The following abbreviations are used in this manuscript:

CUF	Centrifugation-assisted ultrafiltration
GSE	PLE grape seed extract
PLE	Pressurized liquid extraction
STE	PLE grape stem extract
PSTE	PLE grape stem permeate
PGSE	PLE grape seed permeate

## References

[1] Wibisono D.A.S., Saw C.Y., Wu T.Y., Chau C.F. (2025). Advancing industrial food byproduct management: Strategies, technologies, and Progress in waste reduction. Processes.

[2] Zheng S., Huang Z., Dong L., Li D., Hu X., Chen F., Ma C. (2025). Sustainable extraction technology of fruit and vegetable residues as novel food ingredients. Foods.

[3] Todorov S.D., de Almeida B.M., Lima E.M.F., Fabi J.P., Lajolo F.M., Hassimotto N.M.A. (2025). Phenolic Compounds and Bacteriocins: Mechanisms, Interactions, and Applications in Food Preservation and Safety. Mol. Nutr. Food Res..

[4] Rutkowska J., Pasqualone A. (2025). Plant extracts as functional food ingredients. Foods.

[5] Brenes A., Viveros A., Chamorro S., Arija I. (2016). Use of polyphenol-rich grape by-products in monogastric nutrition. A review. Anim. Feed Sci. Technol..

[6] Santos F.T., Goufo P., Santos C., Botelho D., Fonseca J., Queirós A., Costa M.S.S.M., Trinidade H. (2016). Comparison of five agro-industrial waste-based composts as growing media for lettuce: Effect on yield, phenolic compounds and vitamin C. Food Chem..

[7] Tsiapali O.I., Ayfantopoulou E., Tzourouni A., Ofrydopoulou A., Letsiou S., Tsoupras A. (2025). Unveiling the Utilization of Grape and Winery By-Products in Cosmetics with Health Promoting Properties. Appl. Sci..

[8] Nieto J.A., Santoyo S., de Sá M., Baoshan S., Reglero G., Jaime L. (2024). Comprehensive study of sustainable pressurized liquid extractions to obtain bioavailable antioxidant phenolic compounds from grape seed by-products. Processes.

[9] Nieto J.A., Jaime L., Arranz E., Reglero G., Santoyo S. (2017). Winemaking by-products as anti-inflammatory food ingredients. Food Agric. Immunol..

[10] Carrillo J.Á., Arcusa R., Xandri-Martínez R., Cerdá B., Zafrilla P., Marhuenda J. (2025). Impact of Polyphenol-Rich Nutraceuticals on Cognitive Function and Neuroprotective Biomarkers: A Randomized, Double-Blind, Placebo-Controlled Clinical Trial. Nutrients.

[11] Nieto J.A., Fernández-Jalao I., Siles-Sánchez M.D.L.N., Santoyo S., Jaime L. (2023). Implication of the polymeric phenolic fraction and matrix effect on the antioxidant activity, bioaccessibility, and bioavailability of grape stem extracts. Molecules.

[12] Sanz-Buenhombre M., Villanueva S., Moro C., Tomás-Cobos L., Viadel B., Guadarrama A. (2016). Bioavailability and the mechanism of action of a grape extract rich in polyphenols in cholesterol homeostasis. J. Funct. Foods.

[13] Serra A., Macia A., Romero M.-P., Valls J., Blade C., Arola L., Motilva M.-J. (2010). Bioavailability of procyanidin dimers and trimers and matrix food effects in in vitro and in vivo models. Br. J. Nutr..

[14] Jara-Palacios M.J., Gonçalves S., Hernanz D., Heredia F.J., Romano A. (2018). Effects of in vitro gastrointestinal digestion on phenolic compounds and antioxidant activity of different white winemaking byproducts extracts. Food Res. Int..

[15] Toro-Uribe S., López-Giraldo L.J., Alvarez-Rivera G., Ibáñez E., Herrero M. (2019). Insight of Stability of Procyanidins in Free and Liposomal Form under an In Vitro Digestion Model: Study of Bioaccessibility, Kinetic Release Profile, Degradation, and Antioxidant Activity. J. Agric. Food Chem..

[16] Ramos-Pineda A.M., Carpenter G.H., García-Estévez I., Escribano-Bailon M.T. (2019). Influence of chemical species on polyphenol–protein interactions related to wine astringency. J. Agric. Food Chem..

[17] Soares S., Kohl S., Thalmann S., Mateus N., Meyerhof W., De Freitas V. (2013). Different phenolic compounds activate distinct human bitter taste receptors. J. Agric. Food Chem..

[18] Assumpção C.F., Hermes V.S., Pagno C., Castagna A., Mannucci A., Sgherri C., Pinzino C., Ranieri A., Flôres Hickmann S., de Oliveira Rios A. (2018). Phenolic enrichment in apple skin following post-harvest fruit UV-B treatment. Postharvest Biol. Technol..

[19] Villalva M., Jaime L., Aguado E., Nieto J.A., Reglero G., Santoyo S. (2018). Anti-inflammatory and antioxidant activities from the basolateral fraction of Caco-2 cells exposed to a rosmarinic acid enriched extract. J. Agric. Food Chem..

[20] Hamedi F., Mohebbi M., Shahidi F., Azarpazhooh E. (2018). Ultrasound-Assisted Osmotic Treatment of Model Food Impregnated with Pomegranate Peel Phenolic Compounds: Mass Transfer, Texture, and Phenolic Evaluations. Food Bioprocess Technol..

[21] Aryanti P.T.P., Nugroho F.A., Lugito G., Khoiruddin K. (2025). Tight ultrafiltration membranes: Advancing separation technologies for water and wastewater treatment. Sep. Purif. Technol..

[22] Conde E., Díaz Reinoso B., Gonzáles-Muñoz M.J., Moure A., Domínguez H., Parajó J.C. (2013). Recovery and concentration of antioxidants from industrial effluents and from processing streams of underutilized vegetal biomass. Food Public Health.

[23] Li J., Chase H.A. (2010). Applications of membrane techniques for purification of natural products. Biotechnol. Lett..

[24] Cassano A., De Luca G., Conidi C., Drioli E. (2017). Effect of polyphenols-membrane interactions on the performance of membrane-based processes. A review. Coord. Chem. Rev..

[25] Liu D., Vorobiev E., Savoire R., Lanoisellé J.L. (2011). Intensification of polyphenols extraction from grape seeds by high voltage electrical discharges and extract concentration by dead-end ultrafiltration. Sep. Purif. Technol..

[26] Drioli E., Romano M. (2001). Progress and new perspectives on integrated membrane operations for sustainable industrial growth. Ind. Eng. Chem. Res..

[27] Prodanov M., Garrido I., Vacas V., Lebrón-Aguilar R., Dueñas M., Gómez-Cordovés C., Bartolomé B. (2008). Ultrafiltration as alternative purification procedure for the characterization of low and high molecular-mass phenolics from almond skins. Anal. Chim. Acta.

[28] Nieto J.A., Santoyo S., Prodanov M., Reglero G., Jaime L. (2020). Valorisation of Grape Stems as a Source of Phenolic Antioxidants by Using a Sustainable Extraction Methodology. Foods.

[29] Singleton V.L., Orthofer R., Lamuela-Raventós R.M. (1999). Analysis of total phenols and other oxidation substrates and antioxidants by means of Folin-Ciocalteu Reagent. Methods Enzymol..

[30] Re R., Pellegrini N., Proteggente A., Pannala A., Yang M., Rice-Evans C. (1999). Antioxidant activity applying an improved ABTS radical cation decolorization assay. Free Radic Biol. Med..

[31] Brand-Williams W., Cuveleir M.E., Berset C. (1995). Use of a free radical method to evaluate antioxidant activity. LWT-Food Sci. Technol..

[32] Mosmann T. (1983). Rapid colorimetric assay for cellular growth and survival: Application to proliferation and cytotoxicity assays. J. Immunol. Methods.

[33] De Sá M., Justino V., Spranger M.I., Zhao Y.Q., Hanc L., Sun B. (2014). Extraction yields and antioxidant activity of proanthocyanidins from different parts of grape pomace: Effect of mechanical treatments. Phytochem. Anal..

[34] Tabart J., Kevers C., Pincemail J., Defraigne J.O., Dommes J. (2009). Comparative antioxidant capacities of phenolic compounds measured by various tests. Food Chem..

[35] Schaich K.M., Tian X., Xie J. (2015). Reprint of “Hurdles and pitfalls in measuring antioxidant efficacy: A critical evaluation of ABTS, DPPH, and ORAC assays”. J. Funct. Foods.

[36] Tournour H.H., Segundo M.A., Magalhães L.M., Barreiros L., Queiroz J., Cunha L.M. (2015). Valorization of grape pomace: Extraction of bioactive phenolics with antioxidant properties. Ind. Crop. Prod..

[37] Ferreyra S., Torres-Palazzolo C., Bottini R., Camargo A., Fontana A. (2021). Assessment of in-vitro bioaccessibility and antioxidant capacity of phenolic compounds extracts recovered from grapevine bunch stem and cane by-products. Food Chem..

[38] Krasteva D., Ivanov Y., Chengolova Z., Godjevargova T. (2023). Antimicrobial potential, antioxidant activity, and phenolic content of grape seed extracts from four grape varieties. Microorganisms.

[39] Nawaz H., Shi J., Mittal G.S., Kakuda Y. (2006). Extraction of polyphenols from grape seeds and concentration by ultrafiltration. Sep. Purif. Technol..

[40] Neagu E., Roman G.P., Radu G.L. (2010). Antioxidant capacity of some *Symphytum officinalis* extracts processed by ultrafiltration. Rom. Biotechnol. Lett..

[41] Evans P.J., Bird M.R., Rogers D., Wright C.J. (2009). Measurement of polyphenol–membrane interaction forces during the ultrafiltration of black tea liquor. Colloids Surf. A Physicochem. Eng. Asp..

[42] Susanto H., Feng Y., Ulbricht M. (2009). Fouling behavior of aqueous solutions of polyphenolic compounds during ultrafiltration. J. Food Eng..

[43] Kopjar M., Vukoja J., Buljeta I., Ćorković I., Pichler A., Šimunović J. (2023). Formulation and stability of cellulose particles enriched with phenolic acids. Pol. J. Food Nutr. Sci..

[44] Phan A.D.T., Netzel G., Wang D., Flanagan B.M., D’Arcy B.R., Gidley M.J. (2015). Binding of dietary polyphenols to cellulose: Structural and nutritional aspects. Food Chem..

[45] Liu Y., Ying D., Sanguansri L., Cai Y., Le X. (2018). Adsorption of catechin onto cellulose and its mechanism study: Kinetic models, characterization and molecular simulation. Food Res. Int..

[46] Sánchez-Arévalo C.M., Aldegheri F., Vincent-Vela M.C., Álvarez-Blanco S. (2024). Integrated Membrane Process in Organic Media: Combining Organic Solvent Ultrafiltration, Nanofiltration, and Reverse Osmosis to Purify and Concentrate the Phenolic Compounds from Wet Olive Pomace. Int. J. Mol. Sci..

[47] Mejia J.A.A., Ricci A., Figueiredo A.S., Versari A., Cassano A., De Pinho M.N., Parpinello G.P. (2022). Membrane-based operations for the fractionation of polyphenols and polysaccharides from winery sludges. Food Bioprocess Technol..

[48] McDougall G.J., Fyffe S., Dobson P., Stewart D. (2005). Anthocyanins from red wine—Their stability under simulated gastrointestinal digestion. Phytochemistry.

[49] Gonthier M.P., Donovan J.L., Texier O., Felgines C., Remesy C., Scalbert A. (2003). Metabolism of dietary procyanidins in rats. Free. Radic. Biol. Med..

[50] Said I.H., Truex J.D., Heidorn C., Retta M.B., Petrov D.D., Haka S., Kuhnert N. (2020). LC-MS/MS based molecular networking approach for the identification of cocoa phenolic metabolites in human urine. Food Res. Int..

[51] Pereira-Caro G., Gaillet S., Ordóñez J.L., Mena P., Bresciani L., Bindon K.A., Del Rio D., Rounet J.M., Moreno Rojas J.M., Crozier A. (2020). Bioavailability of red wine and grape seed proanthocyanidins in rats. Food Funct..

[52] Lingua M.S., Theumer M.G., Kruzynski P., Wunderlin D.A., Baroni M.V. (2019). Bioaccessibility of polyphenols and antioxidant properties of the white grape by simulated digestion and Caco-2 cell assays: Comparative study with its winemaking product. Food Res. Int..

[53] Chen G.-L., Hu K., Zhong N.-J., Guo J., Gong Y.-S., Deng X.-T., Huang Y.-S., Chu D.-K., Gao Y.-Q. (2013). Antioxidant capacities and total polyphenol content of nine commercially available tea juices measured by an in vitro digestion model. Eur. Food Res. Technol..

[54] Neilson A., Hopf A., Cooper B., Pereira M.A., Bomser J.A., Ferruzzi M.G. (2007). Catechin degradation with concurrent formation of homo- and heterocatechin dimers during in vitro digestion. J. Agric. Food Chem..

[55] Zhu Q.Y., Holt R.R., Lazarus S.A., Ensunsa J.L., Hammerstone J.F., Schmitz H.H., Keen C.L. (2002). Stability of the Flavan-3-ols Epicatechin and Catechin and Related Dimeric Procyanidins Derived from Cocoa. J. Agric. Food Chem..

[56] Krook M.A., Hagerman A.E. (2012). Stability of polyphenols epigallocatechin gallate and pentagalloyl glucose in a simulated digestive system. Food Res. Int..

[57] Laurent C., Besançon P., Caporiccio B. (2007). Flavonoids from a grape seed extract interact with digestive secretions and intestinal cells as assessed in an in vitro digestion/Caco-2 cell culture model. Food Chem..

[58] Tagliazucchi D., Verzelloni E., Bertolini D., Conte A. (2010). In vitro bio-accessibility and antioxidant activity of grape polyphenols. Food Chem..

[59] Patanè G.T., Putaggio S., Tellone E., Barreca D., Ficarra S., Maffei C., Calderaro A., Laganà G. (2023). Catechins and proanthocyanidins involvement in metabolic syndrome. Int. J. Mol. Sci..

[60] Martinez-Gonzalez A.I., Díaz-Sánchez Á.G., Rosa L.A., Vargas-Requena C.L., Bustos-Jaimes I. (2017). Polyphenolic compounds and digestive enzymes: In vitro non-covalent interactions. Molecules.

[61] Konishi Y., Kobayasi S., Shimizu M. (2003). Transepithelial transport of p-coumaric acid and gallic acid in caco-2 cell monolayers. Biosci. Biotechnol. Biochem..

[62] Willengberg I., Michel M., Wonik J., Bartel L.C., Empl M.T., Schebb N.H. (2015). Investigation of the absorption of resvetratrol oligomers in the Caco-2 cellular model of intestinal absorption. Food Chem..

[63] Maier-Salamon A., Hagenauer B., Wirth M., Gabor F., Szekeres T., Jäger W. (2006). Increased transport of resveratrol across monolayers of the human intestinal Caco-2 cells is mediated by inhibition and saturation of metabolites. Pharm. Res..

[64] Manach C., Williamson G., Morand C., Scalbert A., Rémésy C. (2005). Bioavailability and bioefficacy of polyphenols in humans. Review of 97 bioavailability studies. Am. J. Clin. Nutr..

